# Tyrosine-Protein Phosphatase Non-receptor Type 9 (PTPN9) Negatively Regulates the Paracrine Vasoprotective Activity of Bone-Marrow Derived Pro-angiogenic Cells: Impact on Vascular Degeneration in Oxygen-Induced Retinopathy

**DOI:** 10.3389/fcell.2021.679906

**Published:** 2021-05-28

**Authors:** Michel Desjarlais, Pakiza Ruknudin, Maëlle Wirth, Isabelle Lahaie, Rabah Dabouz, José Carlos Rivera, Tiffany Habelrih, Samy Omri, Pierre Hardy, Alain Rivard, Sylvain Chemtob

**Affiliations:** ^1^Department of Ophthalmology, Maisonneuve-Rosemont Hospital Research Center, University of Montréal, Montréal, QC, Canada; ^2^Departments of Pediatrics, Ophthalmology and Pharmacology, Centre Hospitalier Universitaire Sainte-Justine Research Center, Montréal, QC, Canada; ^3^Department of Medicine, Centre Hospitalier de l’Université de Montréal (CHUM) Research Center, Montréal, QC, Canada

**Keywords:** pro-angiogenic cell, vascular degeneration, tyrosine-protein phosphatase non-receptor type 9, oxygen-induced retinopathy, angiogenesis

## Abstract

**Background and Aim:**

Insufficient post-ischemic neovascularization is an initial key step in the pathogenesis of Oxygen-Induced Retinopathy (OIR). During neovascularization, pro-angiogenic cells (PACs) are mobilized from the bone marrow and integrate into ischemic tissues to promote angiogenesis. However, the modulation of PAC paracrine activity during OIR and the specific mechanisms involved remain to be explored. Because Tyrosine-protein phosphatase non-receptor type 9 (PTPN9) is reported to be a negative regulator of stem cell differentiation and angiogenesis signaling, we investigated its effect on PAC activity in the context of OIR.

**Methods and Results:**

In a rat model of OIR, higher levels of PTPN9 in the retina and in bone marrow derived PACs are associated with retinal avascular areas, lower levels of the mobilization factor SDF-1 and decreased number of CD34^+^/CD117^+^/CD133^+^ PACs. PACs exposed *ex vivo* to hyperoxia display increased PTPN9 expression, which is associated with impaired ability of PAC secretome to promote angiogenesis *ex vivo* (choroidal vascular sprouting) and *in vitro* (endothelial cell tubule formation) compared to the secretome of PACs maintained in normoxia. Suppression of PTPN9 (using siRNA) increases VEGF and SDF-1 expression to normalize PAC secretome during hyperoxia, leading to restored angiogenic ability of PAC secretome. Moreover, endothelial cells exposed to the secretome of siPTPN9-treated PACs expressed increased levels of activated form of VEGF receptor 2 (VEGFR2). In the rat model of OIR, intravitreal injection of secretome from siPTPN9-treated PACs significantly reduced retinal vaso-obliteration; this was associated with higher retinal levels of VEGF/SDF-1, and increased recruitment of PACs (CD34^+^ cells) to the retinal and choroidal vessels.

**Conclusion:**

Our results suggest that hyperoxia alters the paracrine proangiogenic activity of BM-PACs by inducing PTPN9, which can contribute to impair post-ischemic revascularization in the context of OIR. Targeting PTPN9 restores PAC angiogenic properties, and provide a new target for vessel integrity in ischemic retinopathies.

## Introduction

Retinopathy of prematurity (ROP) remains a leading cause of visual impairment and blindness in premature neonates worldwide (Rivera et al., 2016). This multifactorial ocular disease is characterized by an initial phase of retinal vascular degeneration resulting in retinal ischemia, which in turn predisposes to excessive pathological intravitreal neovascularization. In ocular ischemic diseases such as ROP, pathological revascularization, also named pathological neovascularization (NV), refers to abnormal and excessive NV from retinal and subretinal vessels into the normally avascular outer retina and subretinal area, unlike the healthy, adequate and coordinated revascularization that occurs following ischemic events (Rivera et al., 2016, 2017a; Desjarlais et al., 2019b).

ROP has been strongly related to high oxygenation (hyperoxia) acting as an initial key element leading to delayed microvascular development of the retina and choroidal involution (Rivera et al., 2016, 2017a; Desjarlais et al., 2019b). At molecular level, several studies have shown that hyperoxia has a deleterious effect by curtailing physiological production of numerous pro-angiogenic factors such as IGF-1, FGF-2, PDGF, SDF-1 and especially VEGF–recognized as the most important growth factor for the maintenance of retinal microvascular development (Rivera et al., 2016, 2017a; Desjarlais et al., 2019b). There has been an emphasis on the development of novel anti-angiogenic therapies to attenuate the excessive neovascularization (Al-Latayfeh et al., 2012). Whereas relatively little is known to either maintain vessel integrity or promote revascularization during the initial phase of ischemia; this know-how could yield approaches to limit subsequent pathological neovascularization. In this regard, angiogenic therapies using stem cell supplementation have recently been shown to promote vascular repair in ischemic animal models (Leeper et al., 2010; Bian et al., 2019).

Many cell types, mostly hematopoietic progenitors (HPCs), display reparative angiogenic properties in ischemic tissues subjected to myocardial infarction, stroke and limb ischemia (Leeper et al., 2010; Choi et al., 2015; Bian et al., 2019). Of these HPCs, bone marrow (BM)-derived proangiogenic cells (PACs), originally described as early outgrowth endothelial progenitor cells (EPCs), have been shown to integrate ischemic tissues and stimulate angiogenesis by secreting angiogenic factors (Choi et al., 2015; Rose et al., 2015; Keighron et al., 2018). Accordingly, systemic or local administration of naïve or reprogrammed PACs or PAC conditioned media, are effective to promote neovascularization in different animal models of hindlimb ischemia (Ranghino et al., 2012; Yu et al., 2015), myocardial infarction (Schuh et al., 2008; Cheng et al., 2013) and ischemic stroke (Fan et al., 2010; Acosta et al., 2019).

Interestingly, in humans and animal models, we and others have reported that pathological conditions and risk factors such as aging (Thum et al., 2007; Groleau et al., 2011), hypercholesterolemia (Desjarlais et al., 2017, 2019a) and diabetes (Loomans et al., 2004) are associated with a reduced number of circulating PACs with impaired PAC activity (Huang et al., 2014) reduced PAC activity contributes in turn to insufficient post-ischemic neovascularization partly via induction of oxidant stress (Imanishi et al., 2008). In diabetic retinopathy, reduced PAC numbers have been observed during the non-proliferative stage; while in the proliferative stage they are markedly increased resulting in aberrant neovascularization (Lois et al., 2014). In ischemic retinopathy induced by diabetes, PACs affected by neurotrophic factors can deregulate and aggravate pathological neovascularization (Liu et al., 2010); a comparable paradigm is observed in laser-induced choroidal neovascularization (Zhang et al., 2011). Hence, depending on the neovascular stage of the condition, PACs can exert different effects. In ROP, a larger number of PACs is detected in circulation, compared to subjects without ROP (Bertagnolli et al., 2017). Whereas, in a model of developmental ischemic retinopathy the role of PACs on retinal and choroidal vascular preservation and/or revascularization remains to be explored; moreover, the specific mechanism responsible for possible PAC dysfunction is unclear.

Protein-tyrosine phosphatases (PTPs) represent a family of 125 PTPs members that can be divided into receptor PTPs and non-receptor cytoplasmic PTPs (Tautz et al., 2013). PTPs can promote or inhibit signal transduction by removing a phosphate group from the tyrosine residues of proteins (Tautz et al., 2013). PTPs are now recognized as key regulators of cellular processes including differentiation, migration, proliferation, death and angiogenesis (Hale et al., 2017). Protein-tyrosine phosphatase-9 (PTPN9 or PTP-MEG2), a cytoplasmic specific PTP member, has been shown to modulate key cellular processes that are essential for angiogenesis and PAC function including VEGF signaling (Corti and Simons, 2017) and stem cell mobilization/maintenance (Hale et al., 2017). We proceeded to investigate the role of PTPN9 in modulating PAC function in a model of ROP. We hereby show that exposure to hyperoxia alters the paracrine proangiogenic activity of BM-PACs, by inducing (at least in part) expression of PTPN9. This results in a drop in PAC numbers that co-localize in vaso-obliterated retinal vessels. PTPN9 knockdown in PACs protects against hyperoxia-induced PAC dysfunction and increases the angiogenic activity of their secretome (conditioned media) by triggering VEGF and SDF-1 generation. Importantly, we show for the first time that intravitreal injection of PTPN9-suppressed PAC secretome prevents microvascular degeneration in the retina and choroid during oxygen-induced retinopathy (OIR). Hence targeting PTPN9 can improve PAC angiogenic activity, and provides a new therapeutic strategy to improve vessel integrity in ischemic retinopathies.

## Materials and Methods

### Animal Care

All animal experimental procedures were performed with strict adherence to the ARVO Statement for the Use of Animals in Ophthalmic and Vision Research and approved by the Animal Care Committee of the Hospital Maisonneuve-Rosemont in accordance with guidelines established by the Canadian Council on Animal Care.

### Identification of Proangiogenic Cells (PACs)/Morphological Characteristics

Bone marrow-derived proangiogenic cells (PACs), also known as “Early outgrowth EPCs” or “circulating angiogenic cells” (CAC) express endothelial markers such lectin, CD31, and VEGFR2, but also hematopoietic stem cell myeloid markers such CD34, C117, and CD133, attesting for a probable monocytic origin (Rose et al., 2015; Desjarlais et al., 2017). In our experiments, spindle-shaped cells were observed, and the vast majority of adherent cells (>90%) were found to uptake DiI-labeled acetylated LDL, bind FITC-labeled lectin, and be positive for the marker CD34 (>80%). We also found that these cells can migrate in response to VEGF stimulation and are capable of incorporating into a network of tubular-like structures when co-cultured with mature endothelial cells. Based on these morphological and functional characteristics and in line with previous studies (Desjarlais et al., 2017, 2019a, 2020a), these cells were characterized and referred to as PACs in this manuscript.

### PACs Isolation From Rat Bone Marrow and Generation of Conditioned Media

Sprague Dawley rat bone marrow mononuclear cells were isolated from the femora and tibiae by flushing the bone marrow cavities using medium 200 (Life technologies) supplemented with 10% fetal bovine serum (FBS, Wisent, St-Jean-Baptiste, QC, Canada), 100 IU/ml penicillin/0.1 mg/ml streptomycin (Wisent) and low serum growth supplement (LSGS; 2% FBS, 3 ng/ml bFGF, 10 mg/ml heparin, 1 mg/ml hydrocortisone, and 10 ng/ml EGF; Life Technologies), and kept on fibronectin-coated plates (Sigma, St. Louis, MO) as previously described (Desjarlais et al., 2017, 2019a, 2020a). After 5 days in culture, non-adherent cells were removed by washing with PBS. Adherent cells were then stained with DAPI (Life Technologies), DIL-low-density lipoprotein (DiI-acLDL, Invitrogen, OR, United States) and FITC-labeled lectin BS-1 (Bandeiraea simplicifolia, Sigma) for 1 h. Bone marrow PACs were characterized as fibronectine adherent cells positive for both DiI-acLDL uptake and lectin binding as previously descripted (Desjarlais et al., 2017, 2019a, 2020a). In addition, adherent cells were also stained with a rabbit CD34 antibody (1:200, ab185732; ABCAM) and secondary antibodies Alexa Fluor 488 anti-rabbit (1:1,000, Life technologies). Quantification was performed by examining several random fields by fluorescence microscopy. To generate PAC conditioned media (CM), naïve PACs or siPTPN9-treated PACs where cultured in a T75 falcon at confluences and stimulated by hypoxia (5% O_2_) for 24 h. The supernatants were then collected, centrifuged briefly, and filtered through 0.22-mm filters (Millipore). For the *in vivo* treatment (intravitreal injection in rat), PAC-CM (siPTPN9-treated PACs) were concentrate (50×) using an Ultra-15 column (Millipore) to reach high concentration of small bioactive molecules. For some experiments, to study the deleterious effects of hyperoxia exposure on PACs activities and their CM, PACs where pre-exposed or not to 80% O_2_ for 6, 24, and 48 h.

### PTPN9 Silencing in PACs

Transfections were carried out using PTPN9 siRNA (validated silencer select, AM4427038, Ambion) or siRNA control scrambled (AM4611, Ambion) using Lipofectamine RNAiMAX Reagent (Thermo Fisher Scientific, ON, Canada) at a concentration of 50 nM according to the manufacturer’s protocol. Briefly, PACs were transfected 24 h before experiments in 6-well plates (for *in vitro* studies) or T75 confluent falcon for the generation of concentrate CM for *in vivo* animal studies. After 6 h, the transfection medium was replaced with antibiotic-free complete M200 medium and cells were subjected or not to hyperoxia (80%) after 24 h of transfection. Transfection efficiency was verified using qRT-PCR and western blot to validate PTPN9 knockdown (suppression levels of 85–90% were observed).

### Oxygen-Induced Retinopathy (OIR) in Rats/Vaso-Obliteration Model

To study the role of PACs in oxygen-induced retinopathy (OIR) and the potential vasoprotective properties of their conditioned media (siPTPN9-treated-PACs) during vascular degeneration, we used a model inducing vaso-obliteration in rats (Desjarlais et al., 2020b). Retinal vaso-obliteration (VO) was induced in Sprague Dawley rat pups subjected to constant hyperoxia (80% O_2_) in chambers controlled by a computer-assisted Oxycycler (BioSpherix, Ltd.) from P5 to P10 as previously descripted (Desjarlais et al., 2020b). Age-matched normoxic control rat pups (NOR) were kept in room air (21% O_2_) throughout the experiment. Thirty minutes before hyperoxia exposure at P5, the OIR pups were anesthetized and intravitreally injected with a single dose of 1 μl of 50× concentrate PAC conditioned media (siPTPN9-treated-PACs media) or PBS used as control. The growth factor free media was collected 24 h after PACs treatment. OIR animals were euthanatized, and retinas collected at P10 for vessel immunostaining (retinal flat mounts and cryosection) and molecular analysis was performed at P10.

### Immunohistochemistry of Retinal and Choroidal Vessels

To analyze retinal vasculature, retinal flat mount dissection was performed on the enucleated eyes fixed in 4% paraformaldehyde for 1 h at room temperature and then stored in PBS until used. The retinas were incubated overnight in 1% Triton X100, 1 mM CaCl2/PBS with the tetramethylrhodamine isothiocyanate–conjugated lectin endothelial cell marker Bandeiraea simplicifolia (1:100; Sigma-Aldrich Corp., St. Louis, MO, United States). Retinas were washed in PBS and mounted on microscope slides (Bio Nuclear Diagnostics, Inc., Toronto, ON, Canada) under coverslips with mounting media (Fluoro-Gel; Electron Microscopy Sciences, Hatfield, PA, United States). Retinas were photographed under an epifluorescence microscope (Zeiss AxioObserver; Carl Zeiss Canada, Toronto, ON, Canada), and the images were merged into a single file using the MosiaX option in the AxioVision 4.6.5 software (Zeiss). For choroidal vasculature, retinal cross-sections were performed. Eyes were collected, dehydrated by alcohol, and embedded in paraffin. Sagittal sections (7 μm thick) were cut by microtome (RM 2145; Leica, Wetzlar, Germany). Posterior eyecups were frozen in optimal cutting temperature medium and stained for choroidal vessels with TRITC-conjugated tetramethylrhodamine isothiocyanate-labeled lectin (Sigma-Aldrich) in the cryosections. Sections were then visualized with an epifluorescence microscope (Eclipse E800; Nikon, Tokyo, Japan). In some experiment, retinal cryosections and flatmount were co-stained (lectin/CD34) by adding a rabbit antibody anti-CD34 (1:200, ab185732; ABCAM) and incubated overnight at 4°C in the blocking solution. Secondary antibodies such as Alexa Fluor 488 anti-rabbit (Life technologies) were used at a dilution of 1:1,000 to detect CD34. Cell nuclei was identified with DAPI labeling. Incubation using rabbit or goat IgG as a primary antibody was conducted as a negative control. The image was split into the three-color channels (RGB Merge/split function) to obtain one image per channel.

### *Ex vivo* Choroidal Angiogenic Sprouting Assay

Angiogenic sprouting capacity of the choroid isolated from rats were assessed as previously descripted (Shao et al., 2013; Desjarlais et al., 2020b; Tomita et al., 2020). Briefly, choroid was isolated from rat pups at P10, sectioned into 1-mm rings, and placed into growth factor–reduced Matrigel (Fisher Scientific, New Hampshire, United States) in 24-well plates and cultured in normoxia (21% O_2_) or hyperoxia (80% O_2_) for 5 days in endothelial growth medium (EGM) used as positive control medium as previously descripted, or in PAC-CM pre-subjected or not to hyperoxia, or siPTPN9-treated PAC-CM. Photomicrographs of individual explants were taken at day 5 using an inverted phase-contrast microscope (AxioObserver; Zeiss), and microvascular sprouting (total area occupied by vessel sprouts excluding the explant) was quantified using Image J.

### Endothelial Cell Culture

Human Retinal Microvascular Endothelial Cells (HRMECs) were purchased from Applied Biological Materials (cat # T4169) and cultured in medium 200 (Life technologies) supplemented with 10% fetal bovine serum (FBS, Wisent, St-Jean-Baptiste, QC, Canada), 100 IU/ml penicillin/0.1 mg/ml streptomycin (Wisent) and low serum growth supplement (LSGS; 2% FBS, 3 ng/ml bFGF, 10 mg/ml heparin, 1 mg/ml hydrocortisone, and 10 ng/ml EGF; Life Technologies). In some experiments, HRMECs were subjected or not to hyperoxia (80%) using oxygen monitoring chambers. HRMECs were grown at 37°C, 5% CO_2_ and 95% air, the medium was changed every 2 days and cells were passaged when they reached 90% confluence; passages 3–6 were used for the experiments.

### Human Retinal Microvascular Endothelial Cells Capillary-Like Tubulogenesis on Matrigel

The angiogenic activity of HRMECs was determined using a Matrigel tube formation assay. Briefly, after transfection and exposure conditions, HRMECs were plated at a density of 30 000 cells/well in 96-well plates precoated with 50 μl of growth factor reduced Matrigel Matrix (Thermo Fisher Scientific, New Hampshire, United States) and cultured at 37°C for 6 h in normoxia or hyperoxia in complete endothelial growth medium (see endothelial cell culture section), or PAC-CM pre-subjected or not hyperoxia, or si-PTPN9-treated-PAC-CM. Capillary-like tubes were observed under a light microscope. Images were obtained at 10× magnification, and all tubes and branching point were counted.

### Incorporation of PACs Into HMREC Tubules

PACs (3,000 cells) pre-labeled with CD34 were co-plated with HUVECs (30 000 cells) in 96-well plates that had been precoated with 50 μl of growth factor reduced Matrigel Matrix (Fisher Scientific, New Hampshire, United States) and cultured at 37°C for 6 h in HMREC negative control media without serum (FBF) and LSGS. Tubular-like structures were photographed and the number of tubes was determined in 6 random fields and compared to PAC-CM treatment or negative control media. A tube was defined as a straight cellular segment connecting two cell masses (nodes). Capillary-like tubes were observed under a fluorescence and light microscope to merged CD34^+^ PACs cells. Images were obtained at 10× magnification, and all tubes and branching point were counted.

### PACs Apoptosis Assay

Pre-transfected PACs (siRNA-PTPN9 or siRNA-control) plated in glass coverslips where subjected to hyperoxia (80% O_2_) for 0, 6, 24, and 48 h to evaluate apoptosis by a Tunel Assay using the *in situ* cell death detection kit (Thermo Fisher Scientific) according to the manufacturing instruction. PAC apoptosis rate was defined as the percentage of Tunel-positive cells (Tunel +) per total dapi-stained PACs nuclei using fluorescence microscopy.

### qRT-PCT Analyses

To quantify PTPN9 and angiogenic factor mRNA levels in PACs and in the rat retinas, total RNA was extracted using RNeasy mini kit (Qiagen) according to the manufacturer’s protocol and was reverse transcribed using iScript-II RT kit (Qiagen) according to manufacturer’s guidelines to generate cDNA. Quantitative real-time PCR reaction was performed using 25 ng of cDNA sample, 2μM of specific primers for the selected mRNAs (Alpha DNA, Montreal, Canada) and Universal SYBR Green Supermix (BioRad). Relative expression (RQ = 2^–ΔΔCT^) was calculated using the instrument detection system ABI Prism 7500 (Applied Biosystems, Foster City, CA, United States) and normalized to b-Actin and GAPDH (Supplementary Table 1).

### Western Blot Analysis

Protein levels of PTPN9, VEGF, SDF-1, P-VEGFR2, and total VEGFR2 were determined by Western blots in the retina of the different groups of rats and in PACs extracts. For total protein extraction, isolated retina were rinsed in PBS, snap-frozen in liquid nitrogen, and stored at −80°C until use. Whole-cell protein extracts were obtained after homogenization of the retina of the different groups of rats in ice cold RIPA buffer (pH = 8) containing 50 mM Tris-HCL, 150 mM NaCl, 5 mM EDTA, 1% Triton 100×, 0.5% sodium deoxycholate, 0.1% SDS with a cocktail of proteases and phosphatases inhibitors (MiniComplete, PhosphoStop and PMSF, Roche, Bâle, Switzerland). PACs were lysed with 50 μl of RIPA lysis buffer per well in 6-well plates, harvested and sonicated. 50 μg of protein per retina homogenate sample and 20 μg of protein per cell lysate sample were separated on an SDS-polyacrylamide gel and electroblotted on nitrocellulose membranes. Non-specific binding sites were blocked with 5% BSA for 1 h. The membranes were probed overnight at 4°C with the following antibodies: mouse monoclonal antibody PTPN9/PTP-MEG2 (1:1,000, MA5-23997, Thermo Fisher Scientific), rabbit antibody VEGF (1:500, sc-152; Santa Cruz Biotechnology, Santa Cruz, CA, United States), rabbit antibody SDF-1 (1:1,000, ab9797; ABCAM), rabbit antibody VEGFR2 (1:1,000, ab39256; ABCAM), rabbit antibody phospho-VEGFR2 Y951 (1:500, ab38473; ABCAM) or GAPDH (1:2,000, ab181602; ABCAM). Membranes were then washed three times for 10 min with TBS-T and incubated with secondary antibodies for 1 h with 1:2,500 horseradish peroxidase (HRP)–conjugated anti-mouse or 1:2,000 HRP-conjugated anti-rabbit secondary antibodies (Millipore). Specific proteins were detected by chemiluminescent reaction (GE Healthcare, Piscataway, NJ) of membranes exposed on LAS-3000 imager. Protein expression was quantified using ImageJ and the results are expressed as density values normalized to the loading control (GAPDH).

### Statistical Analysis

All results are presented as mean ± SEM. Statistical significance was evaluated by a one- or two-way ANOVA followed by a Bonferroni *post hoc* test. A value of *P* < 0.05 was interpreted to denote statistical significance.

## Results

### Phenotypical Characterization of Rat BM-PAC Cultured *ex vivo* and Validation of Their Angiogenic Effects in HMRECs

**FIGURE 1:**
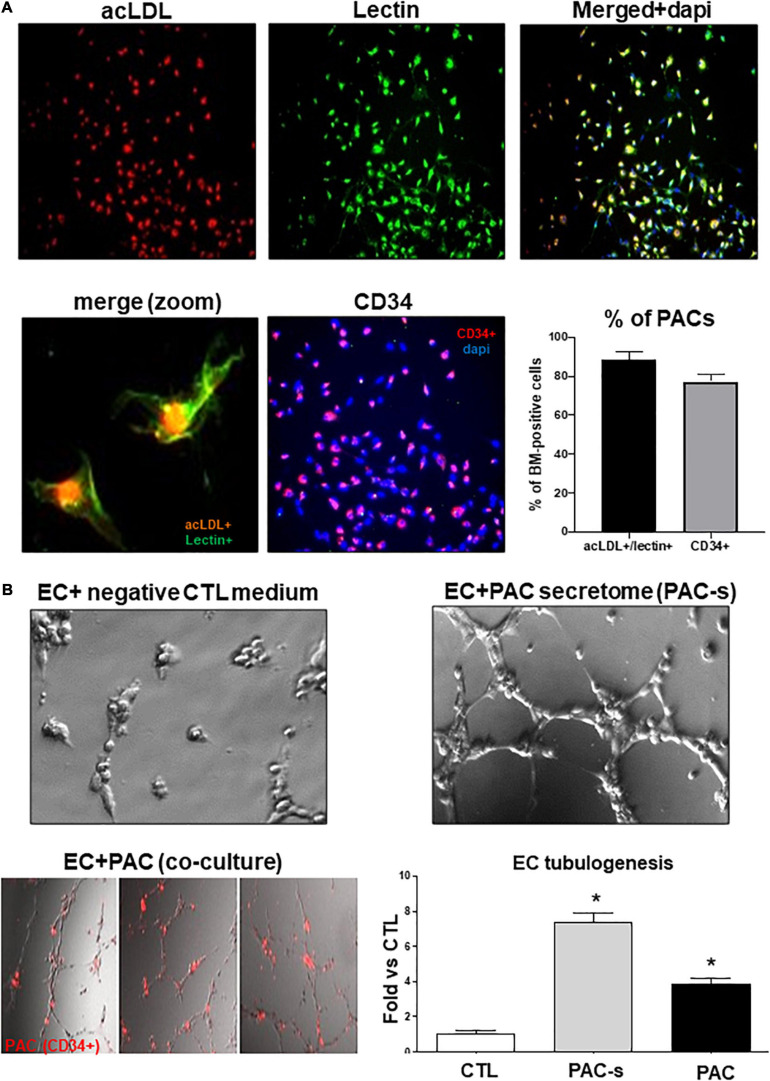
Validation of PAC phenotype and efficacy of PAC-CM to promote retinal endothelial cells angiogenesis. **(A)** Representative pictures of PACs isolated from the bone marrow of rats and cultured *ex vivo* for 4 days. PACs were triple-stained with DAPI (blue), BS-1 lectin-FITC (green), and DiI-acLDL (red) or double stained for DAPI (blue) and CD34 (red). The percentage of isolated cells positive for Lectin^+^/acLDL^+^ or CD34^+^ is shown on the graph. **(B)** Evaluation of the ability of PAC-s to promote HMREC angiogenesis *in vitro* (Matrigel assay) compared to CD34^+^ PACs (red). Culture media without PACs and growth factors was used as a negative control (CTL). Data are mean ± SEM. *P < 0.05 vs. CTL (control). *N* = 3–4 experiments.

To characterize and validate the phenotype of PACs isolated from rat bone marrow, we used a well-described culture method (Desjarlais et al., 2015, 2017, 2020a). Briefly, as summarized in Supplementary Figure 1A, the mononuclear cells isolated from the bone marrow were cultured with endothelial growth medium on a selective adhesion matrix (fibronectin). After 5 days of culture, non-adherent cells were removed and adherent cells that internalize acetylated-LDL (acLDL) and express hematopoietic (CD34, CD133, or CD117) and endothelial (lectin, CD31, or VEGFR2) markers were defined as PACs. As schematized in Supplementary Figure 1B, this population of cells mobilizes into ischemic tissues where they promote neovascularization through secretion of angiogenic growth factors (Choi et al., 2015; Rose et al., 2015; Yu et al., 2015; Keighron et al., 2018). Eighty-five (85%) percent of isolated cells in culture were positive for both lectin and acLDL, and 78% of cells were CD34 immunoreactive (Figure 1A). Isolated PACs adhere to and integrated HMREC capillary-network in matrigel (Figure 1B). PACs and PAC secretome (in conditioned media) effectively induced tubulogenesis in HMRECs (Figure 1B); the latter (7.8-fold increase over control) was more effective than the former (3.8-fold increase over control). Hereon we focused on PAC secretome.

### Hyperoxia Impairs the Angiogenic Properties of PACs by Alterating Paracrine Activity

**FIGURE 2:**
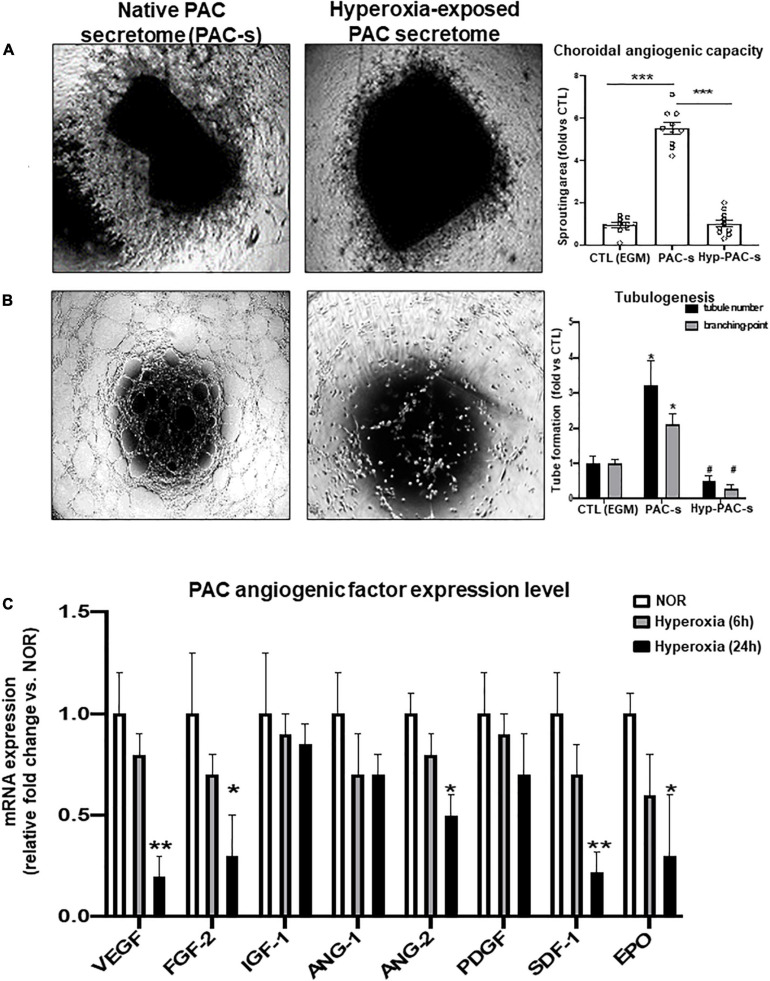
PACs exposed to hyperoxia display reduced paracrine angiogenic activity. **(A,B)** To explore the potential deleterious effect of hyperoxia (80% O_2_) on PAC activity, PACs were exposed for 24 h to hyperoxia (hyp-PAC-s) or normoxia (PAC-s) before harvesting the supernatant (conditioned media). Endothelial growth medium (EGM) was used as a positive control. **(A,B)** Microscopic images and quantification of tubule sprouting in choroid explants after 5 days in culture **(A)** and in HMRECs after 6 h of treatment with the different culture media **(B)**. **(C)** qRT-PCR analyses of angiogenic factor expression in PACs exposed to hyperoxia for 6 and 24 h compared to PAC in normoxic conditions (NOR). Data are mean ± SEM. *P < 0.05 or **P < 0.01 or ***P < 0.001 vs. control (EGM or NOR) or # *P* < 0.05 vs. PAC-s. *N* = 3–4 experiments **(B,C)** or *N* = 10 choroid explant **(A)**.

We evaluated the efficacy of PAC secretome to promote HMREC tubulogenesis and angiogenic sprouting of rat choroidal explant compared to endothelial growth medium [EGM; used as positive control (CTL)], and in this process evaluated effects of *ex vivo* hyperoxia (80% O_2_ for 24 h) on PAC paracrine activities; supernatant was collected at 24 h. PAC secretome-induced angiogenic properties were abrogated by previous exposure of PACs to hyperoxia (Figures 2A,B). These effects were associated with down regulation (at 24 h) of numerous key angiogenic factors, notably FGF-2, ANG-2, EPO, and especially VEGF and SDF-1 (Figure 2C).

### Increased Level of PTPN9 During OIR Correlates With Retinal Avascular Area and Reduced Number of PACs

**FIGURE 3:**
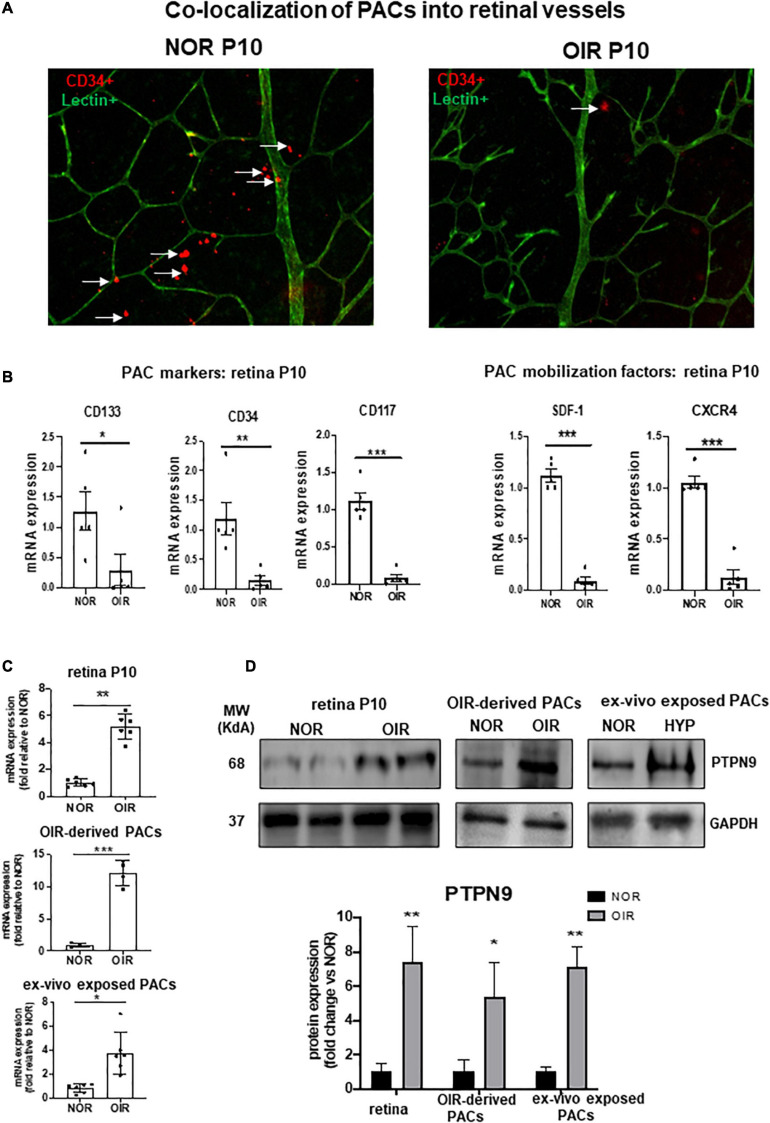
PAC recruitment in retinal vessels is decreased during OIR and associated with increased PTPN9 expression. **(A)** Representative flat mount images of retinal vascularization at P10 showing co-localization of CD34^+^ PACs (red) with lectin positive vessels (green) in OIR-subjected rats vs. control rats kept in normoxic conditions (NOR). **(B)** mRNA expression levels of PAC markers and PAC mobilization factors in the retinas of OIR vs. NOR rats at P10. **(C,D)** mRNA **(C)** and protein **(D)** expression levels of PTPN9 in OIR vs. NOR rats in the retina during OIR at P10, and in PACs isolated from OIR rats or PACs exposed *ex vivo* to hyperoxia. Data are mean ± SEM. *P < 0.05 or **P < 0.01 or ***P < 0.001 vs. control (NOR). *N* = 6–8.

We next evaluated the impact of *in vivo* hyperoxic exposure on PAC number during the vasoobliterative phase of OIR. Diminished vascular density in retinas of rats subjected to OIR (compared to controls) was associated with fewer PACs (CD34^+^ cells) in retina at P10 (Figure 3A). Concordantly, PAC markers (CD34/CD117/CD133) and PAC mobilization factors (SDF-1/CXCR4) were markedly reduced in retinas of OIR rats compared to healthy controls (Figure 3B). Conversely expression of PTPN9 was substantially increased in the retina of OIR-subjected rats, in PACs isolated from hyperoxia-treated rats, and in PACs exposed *ex vivo* to hyperoxia (Figures 3C,D).

### PTPN9 Knockdown Improves the Angiogenic Properties of PACs by Promoting the Paracrine Production of VEGF and SDF-1 During Hypoxia

**FIGURE 4:**
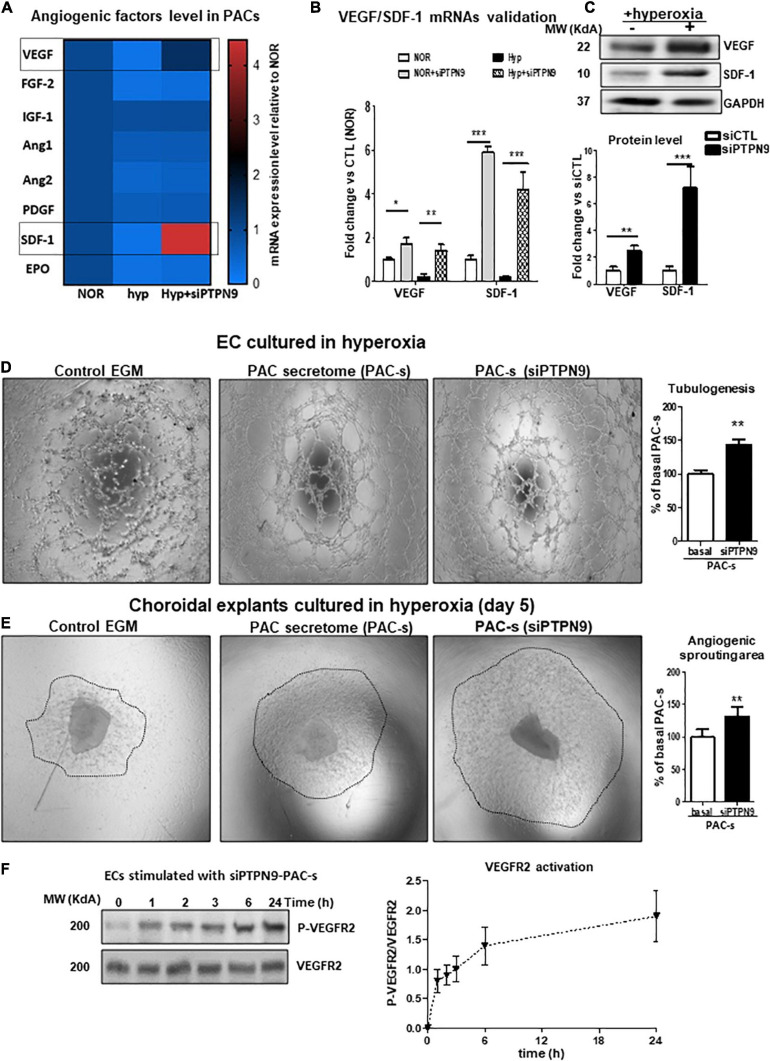
PTPN9 negatively regulates the angiogenic properties of PACs, and PTPN9-silencing protects against hyperoxia-induced PAC dysfunction. **(A)** mRNA analysis of angiogenic factor expression rescue by PTPN9 silencing in PACs subjected to hyperoxia. **(B,C)** qRT-PCR **(B)** and western blot **(C)** validation of the beneficial effects of PTPN9 silencing on SDF-1 and VEGF expression. **(D–F)** Comparison of the effects of siPTPN9-treated-PAC-s, PAC-s and EGM on HMREC tubulogenesis **(D)** and choroidal explant angiogenic sprouting **(E)** in hyperoxic condition. **(F)** Time course analysis of VEGFR2 phosphorylation state in HMRECs treated with siPTPN9-PAC-s under hyperoxic conditions. *N* = 3–4 experiments. *P < 0.05 or **P < 0.01 or ***P < 0.001 vs. respective control (NOR or hyp or basal).

Based on the importance of PTPN9 in stem cell mobilization and maintenance and angiogenic function (Corti and Simons, 2017; Hale et al., 2017), we next investigated the role of PTPN9 on paracrine angiogenic activities of PACs as summarize in Supplementary Figure 2A. Effective PTPN9 knockdown of isolated PACs (siRNA; Supplementary Figures 2A,B) significantly reduced apoptosis upon exposure to hyperoxia (Supplementary Figure 2C). Screening of key angiogenic growth factors in PACs exposed or not to hyperoxia, revealed that PTPN9 suppression can modulate their expression, especially as it applies to VEGF and SDF-1; findings were validated by RT-PCR and protein determination (Figures 4A–C). Correspondingly, PAC secretome of PTPN9-suppressed cells promoted HRMEC tubulogenesis and choroidal sprouting in 24 h hyperoxic conditions (Figures 4D,E), and displayed a time-dependent increase in VEGFR2 activation (increase in phosphorylated VEGFR2; Figure 4F).

**FIGURE 5:**
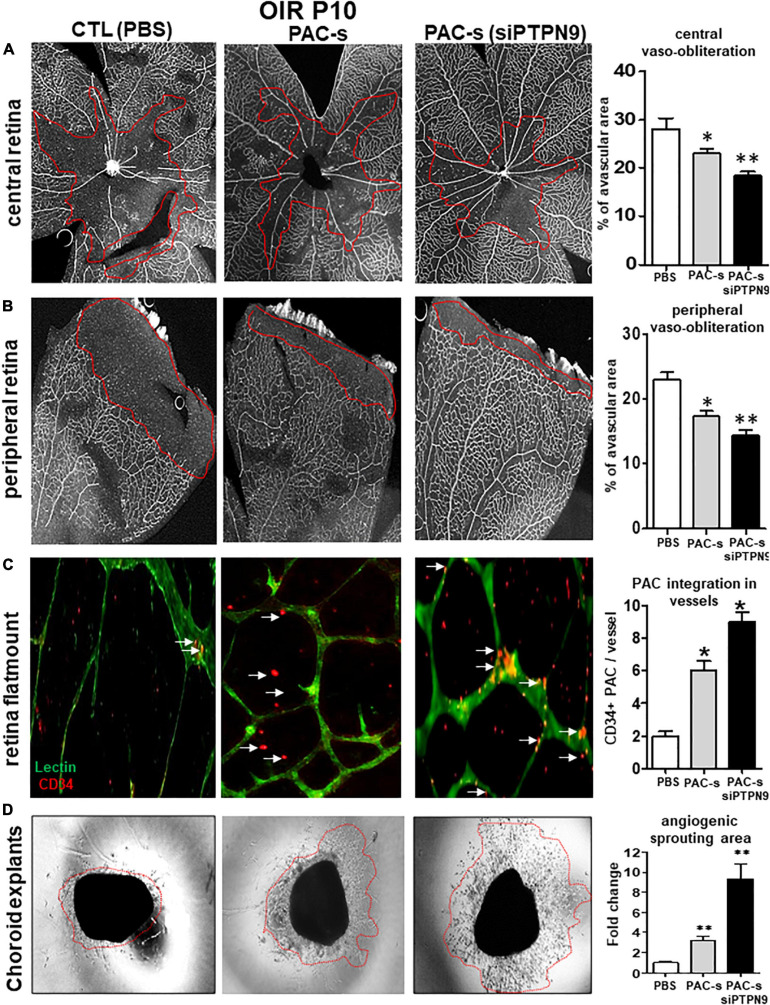
Intraocular injection of siPTPN9-PAC-s improves PAC recruitment and attenuates retinal vaso-oblioteration in rat OIR. **(A)** OIR-subjected rats were treated with a single intravitreal injection (1 μl of 50× concentrate) of PAC-s or siPTPN9-PAC-s or PBS at P5, before exposure to hyperoxia. Animals were then exposed to constant hyperoxia (80% O_2_) until P10. **(A,B)** Representative flat mount images and quantitative analysis of central **(A)** and peripheral **(B)** retinal vaso-obliteration in OIR-subjected rats treated or not with PAC-s or siPTPN9-PAC-s. **(C)** Representative images and analyses of PACs (CD34^+^, orange) co-localizing into vessels (isolectin positive, green)of OIR-subjected rats treated or not with PAC-s or siPTPN9- PAC-s. **(D)** Representative images of choroid explant vascular sprouting (Matrigel) in tissues from OIR animals treated or not with PAC-s or siPTPN9- PAC-s. Data are mean ± SEM. *P < 0.05 or **P < 0.01 vs. PBS (control). *N* = 8–10/group.

**FIGURE 6:**
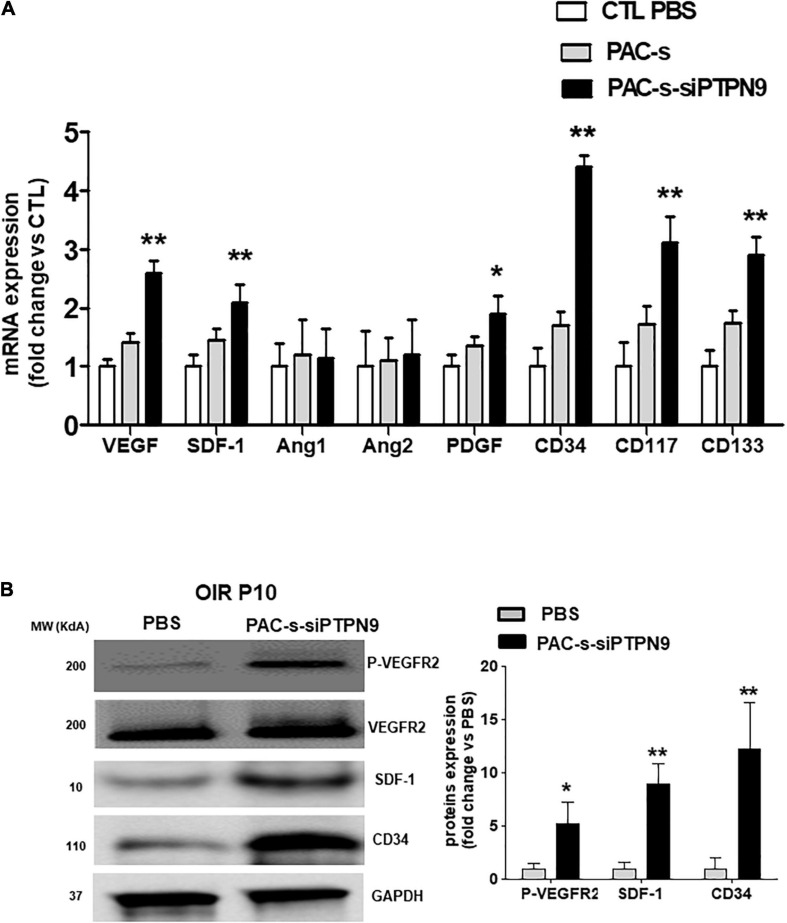
Intraocular injection of siPTPN9-PAC-CM increases the expression level of PAC mobilization factors and stimulates angiogenic signals in the retina of OIR-subjected rats. **(A)** OIR-subjected rats were treated with a single intravitreal injection (1 μl of 50× concentrate) of PAC-s or siPTPN9-PAC-s or PBS at P5, before exposure to hyperoxia. Animals were then exposed to constant hyperoxia (80% O_2_) until P10. **(A,B)** qRT-PCR **(A)** and western blot **(B)** analyses of angiogenic factors, PAC markers, and VEGFR2 activation state (P-VEGFR2) in the retina of OIR-subjected rats treated or not with PAC-s or siPTPN9-PAC-s. Data are mean ± SEM. *P < 0.05 or **P < 0.01 vs. PBS (control). *N* = 4–6 retinas/group for qRT-PCR and a pool of 5 retinas for western blot.

We next tested *in vivo* if PAC secretome of PTPN9-suppressed cells can attenuate retinal vaso-obliteration in rat OIR. We compared effects of intravitreally injected PAC secretome of intact PACs with those of PACs silenced of PTPN9; PACs from OIR are implicitly low in control animals (Figure 3A). [Ideally we intended to suppress PTPN9 of endogenous PACs (using shRNA-encoded lentivirus against PTPN9), but it turned out difficult to target specifically these BM cells]. The secretome of PACs silenced of PTPN9 exhibited highest retinal vascularization (and correspondingly lowest vaso-obliteration) (Figures 5A,B); these PACs integrated more effectively retinal vessels (Figure 5C). Choroids from animals subjected to OIR also exhibited a limited angiogenic ability *ex vivo* (Matrigel), which was restored by silencing PTPN9 in PACs (Figure 5D). Retinas injected with PAC secretome from cells treated with siRNA-PTPN9 displayed increased PAC markers (CD34, CD117, and CD133), suggesting a greater incorporation of PACs in ischemic tissue; these retinas exhibited higher expression levels of major angiogenic factors (including VEGF, phosphorylated VEGFR2, PDGF, and SDF-1; Figures 6A,B).

## Discussion

Throughout life the relationship between neovascularization and hematopoiesis is maintained by a heterogeneous subset of bone marrow-derived hematopoietic progenitor cells that possess the ability to differentiate into proangiogenic cells (PACs) (Rose et al., 2015; Keighron et al., 2018). PACs, previously described as early outgrowth EPCs, have been shown to be mobilized into ischemic tissues where they contribute to vascular repair and neovascularization through paracrine secretion of critical factors involved in angiogenesis and vessel maturation/stabilization including VEGF, FGF, SDF, and PDGF (Rose et al., 2015; Keighron et al., 2018). Several studies have documented reduction in the number and the functional activities of circulating PACs in pathologies associated with insufficient neovascularization after ischemia. For example, in patients with classical cardiovascular risk factors involving oxidative stress and inflammation such as diabetes, dyslipidemia and aging, defective neovascularization in response to ischemia has been linked to impairment in the number and/or the activities of PACs (Loomans et al., 2004; Imanishi et al., 2008; Schuh et al., 2008; Fan et al., 2010; Ranghino et al., 2012; Cheng et al., 2013; Huang et al., 2014; Yu et al., 2015). Interestingly inflammation and oxidative stress is also involved in the physiopathology of ROP (Rivera et al., 2017a,b), a form of vascular degeneration triggered by exposure of immature subjects to hyperoxia. However, the role of PACs in retinal angiogenesis during this period of active developmental repair, and the mechanisms that may be deregulated in this context of vascularization have yet to be investigated.

PACs have been proposed as potential vectors that could be used as “cell therapy” to promote neovascularization and reduce tissue ischemia; their efficacy has been reported in various conditions such as hindlimb ischemia, stroke and diabetic retinopathy (Thum et al., 2007; Schuh et al., 2008; Fan et al., 2010; Ranghino et al., 2012; Cheng et al., 2013; Lois et al., 2014; Choi et al., 2015). Developmental tissues exhibit pronounced repair profiles. Hence, the role of PACs in a model of ROP may differ from that in adult tissues, and is thus particularly relevant for this period of ontogeny. Our study shows that hyperoxia (the principal external factor leading to ROP) reduces PAC recruitment and induces PAC dysfunction; these changes are associated with increased expression of PTPN9, a prominent factor that may regulate paracrine activity of PACs. Accordingly, we hereby show for the first time that by reducing PTPN9 expression in PACs (engineered PACs) improves their angiogenic properties *in vitro*, *ex vivo* and *in vivo* as it attenuates retinal and choroidal vaso-attenuation in OIR. However, the exact mechanism by which PTPN9 modulates the recruitment of PACs into OIR retinas is currently unknown. the fact that we documented increased expression of PTPN9 in bone marrow-derived PACs after OIR could suggest that PTPN9 negatively modulates growth factors (e.g., VEGF, SDF-1) and/or receptors (VEGFR2, CXCR4) that are involved in the mobilization of PACs from the bone marrow. On the other hand, increased recruitment of PACs into retinal vessels following local treatment with the CM of siPTPN9-treated PACs could be related to an “autocrine mechanism” involving increased expression and secretion of angiogenic/chemotactic factors such as VEGF and SDF-1 by PACs.

Our results are consistent with previous studies demonstrating increased efficacy of angiogenic cell-conditioned media compared to the actual cells in promoting revascularization in murine models of hindlimb ischemia (Di Santo et al., 2009; Bhang et al., 2014). Importantly, we found that PACs exposed to hyperoxia display significant reduction of angiogenic activity and fewer of them incorporate in vessels especially in the vaso-attenuated regions, likely due to lower secretion of chemoattractive and angiogenic factors. As per role of oxidant stress in this hyperoxic condition, the latter has been shown to elicit dysfunctions in several hematopoietic cells including PACs, ECFCs, and MSCs (Yao et al., 2006; Case et al., 2008; Porto et al., 2015). Correspondingly, premature newborns exposed to higher oxygen concentrations (than those encountered *in utero*) display decreased numbers and defective activities of circulating PACs (Bertagnolli et al., 2017). In this regard, oxidant stress which participates in major cardiovascular risk factors, can disrupt PAC numbers and their vasoprotective function, leading in some cases to senescence (Huang et al., 2014). For instance, deletion of key antioxidant enzymes such as superoxide dismutase (SOD) has been shown to induce PAC dysfunction (Groleau et al., 2011). Conversely, suppression of pro-oxidative enzymes such as NOX2 can protect the biological function of PACs in pathological conditions (Haddad et al., 2009). Alongside, pharmacological agents exerting antioxidant effects such as statins (Landmesser et al., 2004), and inhibitors of the renin/angiotensin system (Desjarlais et al., 2015), can improve the functional activity of EPCs. Overall, our findings reveal that oxidant stress as encountered during hyperoxia plays a major role in inducing PAC dysfunction. However, the precise mechanisms associated with PAC dysfunction was so far unknown.

An important feature of this study is the evidence showing the role of PTPN9 in suppressing PAC activity in hyperoxia and OIR. PTPN9 has been shown to exert important functions in stem cell differentiation, and is also recognized as an important negative regulator of VEGF signaling (Corti and Simons, 2017; Hale et al., 2017). We found that suppression of PTPN9 in PACs induces their angiogenic ability by increasing production of VEGF and SDF-1, implicated in neovascularization as well as in mobilization of PACs (Odent Grigorescu et al., 2017) and other bone-marrow derived angiogenic cells such as ECFCs (Dragoni et al., 2011; Smadja et al., 2014) and MSCs (Tang et al., 2011), thus rescuing the vascular network and ensued tissue integrity. Our findings agree with those showing that overexpression of PTPN9 induces apoptosis in different tumor cell lines (Wang et al., 2019). In the context of angiogenesis, the effect of PTPN9 seems to be related to dephosphorylation and inactivation of STAT3 (Bu et al., 2014; Kim et al., 2018; Wang et al., 2019), an important survival transcription factor and inhibitor of pro-angiogenic growth factors (Chen and Han, 2008). Thus suppression of PTPN9 in PACs could lead to constitutive activation of STAT3, which has been shown to correlate with the expression of VEGF (Wei et al., 2003). In addition, the JAK2/STAT3 pathway is involved in the SDF-1/CXCR4 interaction promoting mesenchymal stem cell migration in response to the tumor microenvironment (Jin, 2020). Altogether, the recovery of VEGF and SDF-1 expression we observed after silencing PTPN9 in PACs could depend on preservation of STAT3. Other potential mechanisms of PTPN9 knockdown involve EC activates of AKT and Erk (Qu et al., 2019), two major signaling pathways that promote cell migration, proliferation and survival (Cao et al., 2019). However, several other mechanisms could also be involved, and the present study did not identify the exact(s) mechanism(s) by which PTPN9 modulates the expression of angiogenic factors (i.e., VEGF and SDF-1) in PACs.

**FIGURE 7:**
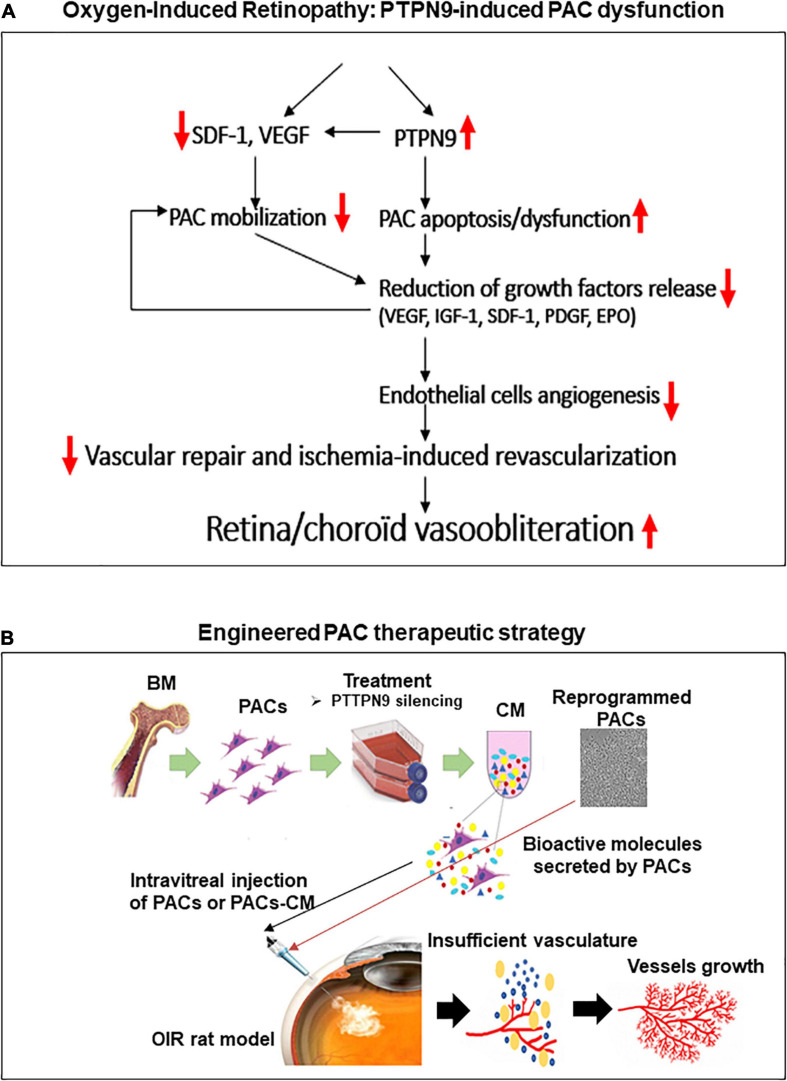
Schematic summary of the study. Proposed mechanisms for the negative regulation of PAC function by PTPN9 during OIR **(A)**, and vaso-protective potential of PAC-based therapy to improve retinal neovascularization **(B)**.

In summary, we report that hyperoxia impairs the recruitment and paracrine proangiogenic activities of BM-PACs through induction of PTPN9, which curtails cell migration and EC proliferation, thus compromising retinal vascularization in OIR; a schematic diagram displaying role of PTPN9 in PACs involved in ocular vascularization is presented in Figure 7. Unlike current anti-VEGF therapies inhibiting pathological NV in the second (late) phase of ROP when vascular damage is already in place, this study also suggests that a protective angiogenic strategy aimed at promoting early physiological NV using PAC secretome could therapeutically limit subsequent pathological NV. Targeting PTPN9 in PACs restores their number and function, providing an unprecedented strategy for vessel integrity and revascularization in ischemic retinopathies.

## Data Availability Statement

The original contributions presented in the study are included in the article/Supplementary Material, further inquiries can be directed to the corresponding author/s.

## Ethics Statement

All animal experimental procedures were performed with strict adherence to the ARVO Statement for the Use of Animals in Ophthalmic and Vision Research and approved by the Animal Care Committee of the Hospital Maisonneuve-Rosemont in accordance with guidelines established by the Canadian Council on Animal Care.

## Author Contributions

MD and SC conceived and designed the study. MD wrote the initial draft of the manuscript and figures, directed and planned the experiments, and analyzed the data. AR and SC revised the manuscript. MD performed the experiments, assisted by MW, IL, PR, JR, SO, and TH. AR, PH, and SC provided expert advices. All authors contributed to the article and approved the submitted version.

## Conflict of Interest

The authors declare that the research was conducted in the absence of any commercial or financial relationships that could be construed as a potential conflict of interest.

## Supplementary Material

Supplementary Figure 1 Overview of the methods used to isolate rat BM-PACs, and schematic overview of PAC paracrine pro-angiogenic actions in ischemic tissues. **(A,B)** PAC isolation, culture and identification **(A)** and illustration of the paracrine angiogenic activities of PACs on local endothelial cells after homing in ischemic tissues **(B)**.

Supplementary Figure 2 PTPN9 knockdown confirmation in BM-PACs, and PTPN9 effect on hyperoxia-induced apoptosis. **(A)** Schematic summary of the experimental approach to explore PTPN9 regulatory function on PAC activities. PACs were pre-transfected or not with 50 nM of scrambled siRNA (control) or a siRNA targeting PTPN9 (siPTPN9), and then subjected or not to hyperoxia (80% O_2_) for 6–48 h. **(B)** Validation of PTPN9 mRNA and protein silencing in PACs using qRT-PCR and western blot analyses. **(C)** Cytoprotective effects of PTPN9 silencing (siPTPN9) in hyperoxic conditions as assessed by Tunnel assay. Representative images and quantitative analyses of apoptosis (red apoptotic cells; blue nuclei) in PACs pre-treated with siPTPN9 or siCTL before exposure to hyperoxia for 6, 24, and 48 h.

Supplementary Figure 3 Intraocular injection of siPTPN9-PAC-CM reduced the choroidal involution of OIR-subjected rats. **(A,B)** Sub-retina showing the effects of siPTPN9-PAC-s on choroidal vessels (green) and PACs (CD34^+^ cells, orange) **(A)**, and on the choroidal vascular involution (vessels in red) **(B)** of OIR-subjected rats treated or not with siPTPN9- PAC-s. Data are mean ± SEM. **P* < 0.05 or ***P* < 0.01 vs. PBS (control). *N* = 4–6/group.

Supplementary Table 1 List of primers used for real time RT-PCT.
