# Supplementary figures and images for: Tyrosine-Protein Phosphatase Non-receptor Type 9 (PTPN9) Negatively Regulates the Paracrine Vasoprotective Activity of Bone-Marrow Derived Pro-angiogenic Cells: Impact on Vascular Degeneration in Oxygen-Induced Retinopathy
Source: Front Cell Dev Biol. 2021 May 28;9:679906. doi: 10.3389/fcell.2021.679906 (PMC8194284; doi:10.3389/fcell.2021.679906)

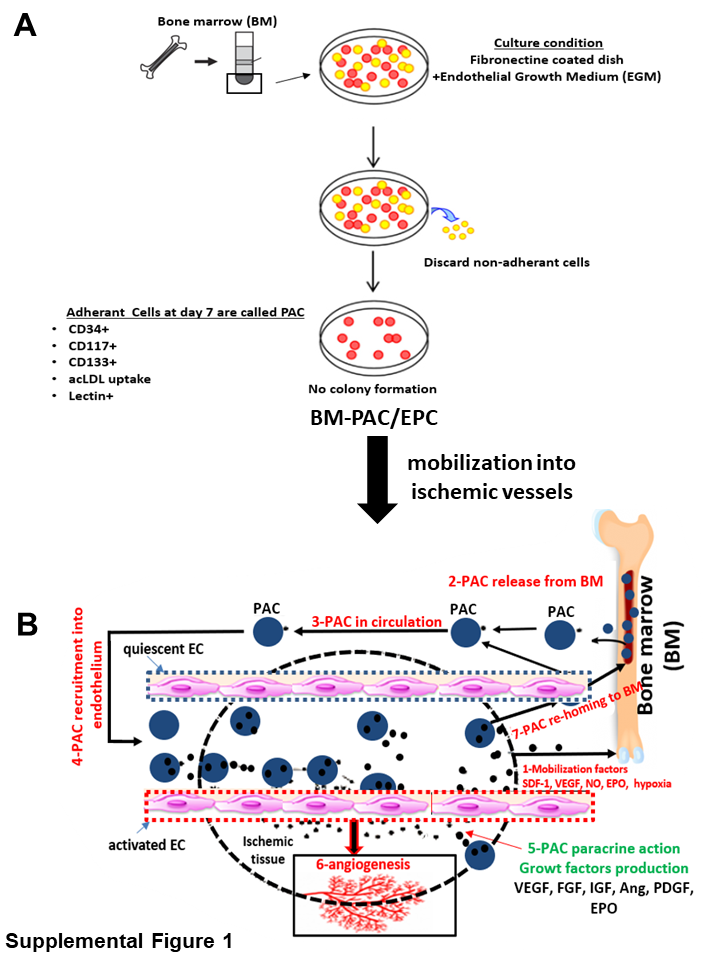

Supplement: Supplementary Figure 1 — Overview of the methods used to isolate rat BM-PACs, and schematic overview of PAC paracrine pro-angiogenic actions in ischemic tissues. (A,B) PAC isolation, culture and identification (A) and illustration of the paracrine angiogenic activities of PACs on local endothelial cells after homing in ischemic tissues (B). [file Image_1.TIF]

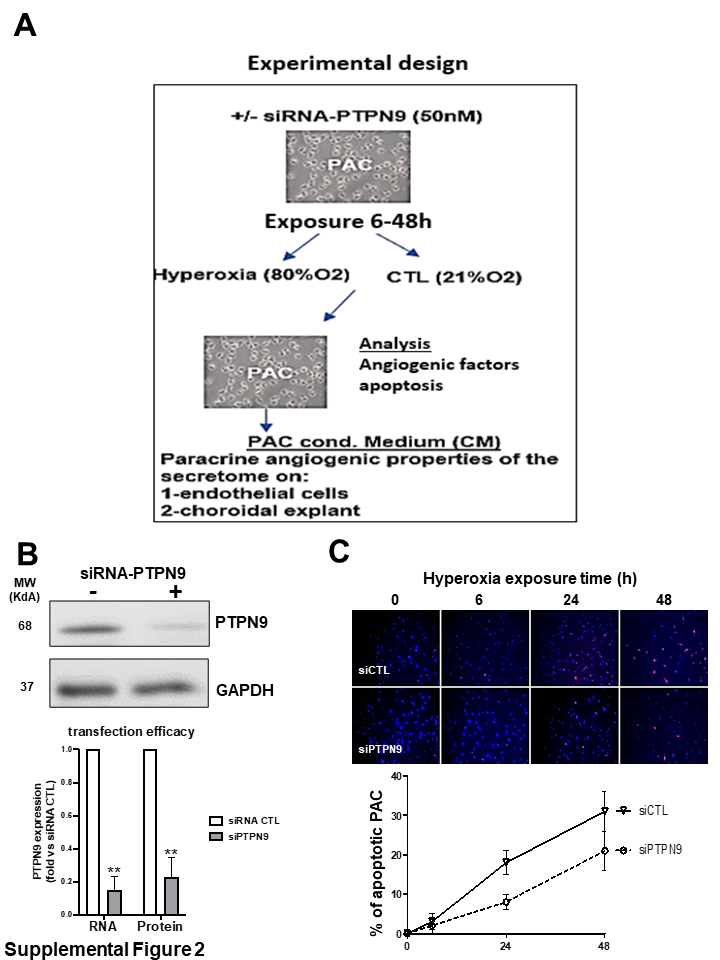

Supplement: Supplementary Figure 2 — PTPN9 knockdown confirmation in BM-PACs, and PTPN9 effect on hyperoxia-induced apoptosis. (A) Schematic summary of the experimental approach to explore PTPN9 regulatory function on PAC activities. PACs were pre-transfected or not with 50 nM of scrambled siRNA (control) or a siRNA targeting PTPN9 (siPTPN9), and then subjected or not to hyperoxia (80% O2) for 6–48 h. (B) Validation of PTPN9 mRNA and protein silencing in PACs using qRT-PCR and western blot analyses. (C) Cytoprotective effects of PTPN9 silencing (siPTPN9) in hyperoxic conditions as assessed by Tunnel assay. Representative images and quantitative analyses of apoptosis (red apoptotic cells; blue nuclei) in PACs pre-treated with siPTPN9 or siCTL before exposure to hyperoxia for 6, 24, and 48 h. [file Image_2.TIF]

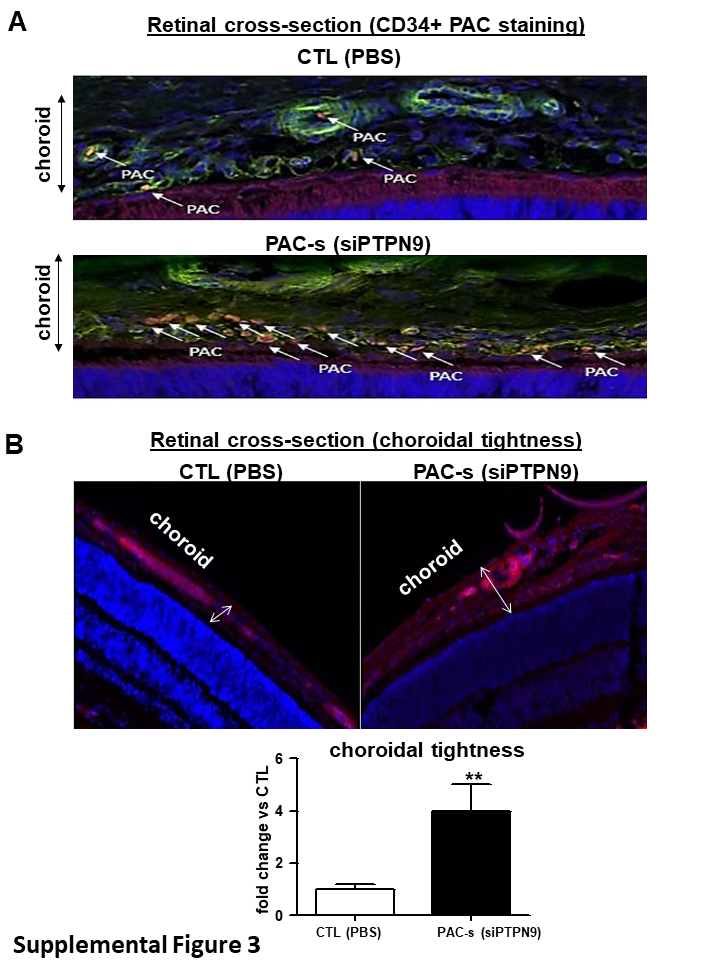

Supplement: Supplementary Figure 3 — Intraocular injection of siPTPN9-PAC-CM reduced the choroidal involution of OIR-subjected rats. (A,B) Sub-retina showing the effects of siPTPN9-PAC-s on choroidal vessels (green) and PACs (CD34+ cells, orange) (A), and on the choroidal vascular involution (vessels in red) (B) of OIR-subjected rats treated or not with siPTPN9- PAC-s. Data are mean ± SEM. *P < 0.05 or **P < 0.01 vs. PBS (control). N = 4–6/group. [file Image_3.TIF]
