# Supplementary material for: Tyrosine-Protein Phosphatase Non-receptor Type 9 (PTPN9) Negatively Regulates the Paracrine Vasoprotective Activity of Bone-Marrow Derived Pro-angiogenic Cells: Impact on Vascular Degeneration in Oxygen-Induced Retinopathy
Source: Front Cell Dev Biol. 2021 May 28;9:679906. doi: 10.3389/fcell.2021.679906 (PMC8194284; doi:10.3389/fcell.2021.679906)
Supplement: Supplementary Table 1 — List of primers used for real time RT-PCT. [file Table_1.PDF]

	Foward	Reverse
ANG-1	TGCAAATGTGCCCTCATGTTA	TCCCGCAGTATAGAACATTCCA
ANG-2	CTGCACAGCATTGGACACGTA	TCCTCCTGCCAGAGATGGAC
β-Actin	GTGGGCCGCACAAGGCACCAA	CTCTTTGATGTCACGCACGA
CD117	ACATCGCCAGAGCCAACG	ATCCACTTTAATTTCGGGTCAA
CD133	GGACCCATTGGCATTCTC	CAGGACACAGCATAGAATAATC
CD34	GTCACACTGCCTACTACTTC	TCCTCGGATTCCTGAACAT
CXCR4	GCCATGGCTGACTGGTACTT	GATGAAGGCCAGGATGAGAA
EPO	CTGTATCATGGACCACCTCGG	TGAAGCACAGAAGCTCTTCGG
FGF-2	CATCAAGCTACAACTTCAAGCAGAA	GCCAGTAATCTTCCATCTTCCTTC
GAPDH	AGCCACATCGCTCAGACACC	GCGCCCAATACGACCAAA
IGF-1	CTTTGCGGGGCTGAGCTGGT	CTTCAGCGAGCAGTACA
PDGF	GGAGGAAGAGAAGCATCGAGG	CGACCTGACTCCGAGGAATCT
PTPN9	CCTGCCTTAGACTGGGACT	TTCGCTTTGTTAGCTTCACT
SDF-1	ATGAACGCCAAGGTCGTGGTC	TGGCTGTTGTGCTTACTTGTTT
VEGF	TTGCTGCTCTACCTCCACCAT	TCTGCCCTCCTCCTTCTGC

Supplemental Table 1. List of primers used for real time RT-PCR.
